# Co-inoculation Effect of Rhizobia and Plant Growth Promoting Rhizobacteria on Common Bean Growth in a Low Phosphorus Soil

**DOI:** 10.3389/fpls.2017.00141

**Published:** 2017-02-07

**Authors:** Hezekiah Korir, Nancy W. Mungai, Moses Thuita, Yosef Hamba, Cargele Masso

**Affiliations:** ^1^Department of Crops, Horticulture and Soils, Egerton UniversityNjoro, Kenya; ^2^International Institute of Tropical AgricultureNairobi, Kenya; ^3^Department of Molecular Biology and Biotechnology, Pan African University Institute of Basic Science, Technology and Innovation, Jomo Kenyatta University of Agriculture and TechnologyNairobi, Kenya

**Keywords:** co-inoculation, *Phaseolus vulgaris* L., *Paenibacillus polymyxa*, *Bacillus megaterium*, rhizobia

## Abstract

Nitrogen (N) fixation through legume-*Rhizobium* symbiosis is important for enhancing agricultural productivity and is therefore of great economic interest. Growing evidence indicates that other soil beneficial bacteria can positively affect symbiotic performance of rhizobia. Nodule endophytic plant growth promoting rhizobacteria (PGPR) were isolated from common bean nodules from Nakuru County in Kenya and characterized 16S rDNA partial gene sequencing. The effect of co-inoculation of *rhizobium* and PGPR, on nodulation and growth of common bean (*Phaseolus vulgaris* L.) was also investigated using a low phosphorous soil under greenhouse conditions. Gram-positive nodule endophytic PGPR belonging to the genus Bacillus were successfully isolated and characterized. Two PGPR strains (*Paenibacillus polymyxa* and *Bacillus megaterium*), two rhizobia strains (IITA-PAU 987 and IITA-PAU 983) and one reference rhizobia strain (CIAT 899) were used in the co-inoculation study. Two common bean varieties were inoculated with *Rhizobium* strains singly or in a combination with PGPR to evaluate the effect on nodulation and growth parameters. Co-inoculation of IITA-PAU 987 + *B. megaterium* recorded the highest nodule weight (405.2 mg) compared to IITA-PAU 987 alone (324.8 mg), while CIAT 899 + *B. megaterium* (401.2 mg) compared to CIAT 899 alone (337.2 mg). CIAT 899 + *B. megaterium* recorded a significantly higher shoot dry weight (7.23 g) compared to CIAT 899 alone (5.80 g). However, there was no significant difference between CIAT 899 + *P. polymyxa* and CIAT 899 alone. Combination of IITA-PAU 987 and *B. megaterium* led to significantly higher shoot dry weight (6.84 g) compared to IITA-PAU 987 alone (5.32 g) but no significant difference was observed when co-inoculated with *P. polymyxa*. IITA-PAU 983 in combination with *P. polymyxa* led to significantly higher shoot dry weight (7.15 g) compared to IITA-PAU 983 alone (5.14 g). Plants inoculated with IITA-PAU 987 and *B. megaterium* received 24.0 % of their nitrogen demand from atmosphere, which showed a 31.1% increase compared to rhizobium alone. Contrast analysis confirmed the difference between the co-inoculation of rhizobia strains and PGPR compared to single rhizobia inoculation on the root dry weight. These results show that co-inoculation of PGPR and Rhizobia has a synergistic effect on bean growth. Use of PGPR may improve effectiveness of *Rhizobium* biofertilizers for common bean production. Testing of PGPR under field conditions will further elucidate their effectiveness on grain yields of common bean.

## Introduction

Nitrogen (N) and phosphorus (P) are among the most limiting nutrients for plant growth. Phosphorus is generally deficient in most of the soils due to its ready fixation (Collavino et al., 2010). Inadequate P restricts root growth, the process of photosynthesis, translocation of sugars, and other such functions, which directly or indirectly influence nitrogen fixation by legume plants (Olivera et al., 2004).

The replenishment of N and P nutrients is mostly done through application of inorganic fertilizer to the soil. However, prices of nitrogen and phosphatic fertilizers have increased, particularly in developing countries. Therefore, it is very challenging for farmers to supplement N and P fertilizers in the soil to avoid the nutrient deficiencies. Given the reported negative environmental impacts of chemical fertilizers and increasing costs, utilization of plant growth promoting rhizobacteria (PGPR), and rhizobia is advantageous for sustainable agricultural practices. Thus, one area of increasing interest is the use of microorganisms with the ability to solubilize mineral and organic P (Khan et al., 2006; Fernández et al., 2007; Shiri-Janagard et al., 2012) or to fix nitrogen (Uribe et al., 2012). The association between PGPR and plant roots plays a key role in P nutrition in many agroecosystems, particularly in P-deficient soils (Goldstein, 2007; Jorquera et al., 2008).

The inoculation of plants with selected PGPR to increase native population can mobilize P from poorly available sources and therefore improve plant nutrition (Richardson et al., 2009; Guiñazu et al., 2010). Increased growth and P uptake have been reported for *Paenibacillus polymyxa* and *Bacillus megaterium* in tomato (Ei-Yazeid and Abou-Aly, 2011). Similarly, legume growth and yields have been shown to increase with inoculation with Rhizobia. Co-inoculation with P-solubilizing bacteria and Rhizobium stimulated plant growth more than their separate inoculations (Morel et al., 2012; Walpola and Yoon, 2013). Bai et al. (2003) reported that co-inoculation of Bacillus strains in soybean plants with *Bradyrhizobium japonicum* provided the largest increases in nodule number, nodule weight, shoot weight, root weight, total biomass, total nitrogen, and grain yield. Results by Tariq et al. (2012) showed that non-rhizobial plant growth promoting bacteria improve nodulation and grain yield of the legumes upon co-inoculation with crop specific rhizobia. Remans et al. (2007) also reported an increased ability of *Rhizobium* isolates nodulation on bean plants as the result of phosphate solubilizing bacteria co-application. Silva et al. (2007) found specific nodulation stimulus and increase root dry matter in *Vigna unguiculata* co-inoculated with *Bradyrhizobium* sp. and *Paenibacillus polymyxa* Loutit (L) and *Bacillus* sp. (LBF-410). Similarly, endophytic plant growth promoting bacteria and nitrogen-fixing Rhizobium species were found to work in synergy to promote nitrogen fixation efficiency in Lentils (Veena and Poonam, 2011; Saini and Khana, 2012).

The PGPR which increases the efficiency of the Rhizobium species in one legume does not necessarily do the same in other legumes. For example, the strain *Bacillus* sp. CECT 450 although it increased nodulation in common bean when co-inoculated with *Rhizobium tropici* CIAT 899, it reduced nodulation in soybean when co-inoculated with *Bradyrhizobium japonicum* USDA 110 strain (Camacho et al., 2001). Similarly, in a study by Elkoca et al. (2010) it was noted that except for *Bacillus subtilis* strain OSU-142 + *B. megaterium* strain M-3, inoculation with dual and triple mixtures with *Rhizobium*, OSU-142, and M-3 had no significant effect on common bean yield compared with single inoculations of these bacteria. The variable responses to co-inoculation underscores the need to identify appropriate combinations of rhizobia strain and PGPR for particular sites to enhance growth of common bean.

In Kenya, much emphasis in bio fertilization of legumes has been put on rhizobia inoculation, particularly for common bean. Importantly, in Kenya, information is scanty regarding isolation of native root endogenous PGPR, the role of PGPR in phosphorus bioavailability, growth promotion and also their interaction with rhizobia in common bean. The present study was therefore designed to (1) isolate and identify PGPR from common bean root nodules and (2) evaluate the effect of co-inoculation of *PGPR* and Rhizobia on growth and nodulation of common beans in a low P soil.

## Materials and Methods

### Isolation and Identification of PGPR

To obtain the native PGPRs in the root nodules of common bean, trapping was done from different soils and subsequently isolated in the laboratory. Extraction of DNA, PCR, and sequencing was done to identify the PGPR isolates.

#### Trapping of PGPR from the Soil

The experiment was conducted using soils from five locations (Rongai, Bahati, Ngata, Egerton, and Lare) in Nakuru County to trap PGPR from the soil using common bean (Tasha variety-Lyamungu 85) as a trap crop. Soils were obtained from fields in which common bean had recently been cultivated. Soil was sampled from different points in the farm and mixed to obtain a composite sample. The soils were air-dried and sieved through 2 mm sieve. Two kilograms of each soil were weighed into white, perforated plastic pots (volume = 2.5 L), and placed on plastic plates. The trial were laid out following a completely randomized design (CRD) with three replicates (5 soils × 3 replicates = 15 pots).

Seeds were surface-sterilized by soaking in 3.5% NaOCl solution for 5 min, then thoroughly washed with sterile, distilled water. Three pre-selected healthy seeds of uniform size were then planted per pot, and thinned to one plant per pot of comparable height and vigor between 1 and 2 weeks after planting. Plants were watered daily using distilled water, and twice daily (if necessary) during later growth stages to avoid water stress and to maintain the soil at field capacity.

A standard nutrient solution containing macronutrients K, Mg, Ca, and S, and micronutrients Mn, Zn, Cu, B, Mo, and Co was prepared at concentrations of 750 mg K, 270 mg Ca, 165 mg Mg, 60 mg S, 36 mg Mn, 1.5 mg Zn, 0.6 mg Cu, 0.9 mg B, 0.15 mg Mo, and 0.15 mg Co L^-1^ (Broughton and Dilworth, 1970). Then 10 ml of the standard nutrient solution pot^-1^ was applied at planting and at 3 weeks after planting, respectively. Crops were grown under greenhouse conditions until R2 stage of growth (7–8 weeks).

At harvest, shoots were cut using a clean, sharp knife at 1 cm above the soil surface. Thereafter, the pots were emptied on a 2 mm sieve and soil was washed gently to isolate the roots and nodules. Nodule samples were taken and stored at –20°C.

#### Laboratory Isolation of PGPR Strains from Nodules

The PGPR strains were isolated from crushed nodules preserved in glycerol and stored at –20° C by streaking onto YEMA plates (Vincent, 1970). Purity of colonies was checked for by repeated streaking on YEM plates and by microscopic examination of living cells. Microscopic observations were performed to investigate some characteristics of the isolates such as shape and gram reaction. Catalase test was also carried out using 24 h old bacterial cultures whereby a single bacterial colony was placed on glass slide and a drop of 30% hydrogen peroxide (H_2_O_2_) added. Appearance of gas bubbles indicated the presence of catalase enzymes in the bacteria. A pure culture was grown in YEM and used for DNA extraction.

#### DNA Extraction from the Isolates

DNA was extracted from seven isolates out of the 19 morphotypes. Selection of various morphotypes was performed on the basis of size, shape, color, and elevation of colonies. Seven of the isolates that were morphologically similar, gram positive and catalase positive showing positive results for the PGPR were used for further characterization. DNA extraction was done as described by Wilson (1987). Liquid culture from the isolation step (1.2 ml) was centrifuged for 5 min at 13000 rpm at room temperature. The supernatant was poured out and the pellet suspended by adding 500 μl of TE 1 X. The suspension was then centrifuged for 5 min at 13000 rpm at room temperature and the supernatant poured out. The pellet was re-suspended in 540 μl of TE 5 X and incubated for 15 min at 70°C. Two microliters of proteinase K, 30 μl of 10% SDS (w/v) were added and incubated for 15 min at 70°C. Six hundred microliters of phenol:chloroform:isoamylalcohol 25:24:1 (v/v/v) was added and centrifuged at 13000 rpm for 5 min at room temperature. The supernatant was transferred to a clean tube and extracted with an equal volume of chloroform:isoamylalcohol 24:1 (v/v) to remove residual phenol. It was centrifuge for 5 min at 13000 rpm at room temperature, and the supernatant transferred into another clean Eppendorf tube and the volume noted. The DNA was precipitated by the addition of 100 μl of ice-cold isopropanol and incubated overnight at -20°C. The precipitated DNA was centrifuged for 5 min at 13000 rpm at 4°C, the supernatant poured out the pellet cleaned with 500 μl of 70% Ethanol. It was then centrifuge for 10 min at 13000 rpm at 4°C and the supernatant poured out. The pellet was air-dried and then dissolved in 50 μl double distilled water and stored at -20°C for further analysis (Compro-II, 2014).

#### Molecular Characterization of the PGPR Strains

The 16S-23S rDNA intergenic region was amplified through PCR. Primers used for amplification were 27F (5′AGAGTTTGATCCTGGCTCAG3′) and 1492R (5′TACGGCTACCTTGTTACGACTT 3′; Lane, 1991). The PCR mix for one sample included: PCR master 12.5 μl, Forward primer 27F 1 μl, Reverse primer 1 μl, sterile distilled water 7.5 μl, and 3 μl DNA template. Amplification was performed in a Bio-Rad PCR system thermal cycler adjusted to the following program: initial denaturation for 5 min at 94°C, 35 cycles of denaturation for 30 s at 94°C, annealing for 30 s at 58°C, extension for 30 s at 72°C, and final extension for 7 min at 72°C. The PCR products were visualized by electrophoresis of 3 μl of the amplified DNA on 1% (w/v) horizontal agarose gel (SIGMA^®^) in TBE buffer (1.1 w/v Tris-HCL; 0.1% w/v Na_2_EDTA 2H_2_O; 0.55% w/v Boric acid), pre-stained with 3.5 μl of ethidium bromide. The gel was photographed under UV illumination with Gel Doc (BIO-RAD) Software (USA). Products with a single band were selected as suitable for purification.

#### Purification of PCR Product and Sequencing

GeneJET PCR Purification Kit was used to purify the DNA. This kit includes binding buffer, washing buffer, and elution buffer. The PCR product was mixed well with binding buffer in a ratio of 1:1, centrifuged for 30 s at 13000 rpm and flow-through was discarded. Then, 700 μl of washing buffer were added each to the mixture and centrifuged for 30 s at 13000 rpm and flow-through was discarded. Then the PCR product was centrifuged for 30 s at 13000 rpm to get rid of all the ethanol. Finally, 50 μl of elution buffer was added, centrifuged for 1 min at 13000 rpm and purified PCR products were collected. The purified PCR product was submitted for sequencing at the Segolip unit of the BeCA hub where Sanger Sequencing was applied using Big Dye Terminator^®^ and cycle sequencing kit used was Big Dye Terminator v3.1. Fluorescence-based PCR run on Applied Biosystems thermal cyclers (GeneAmp^®^ 9700 system) and Purified Cycle Sequencing reactions electrophoresed on ABI 3730 – 48 Capillary Genetic Analyzer. Base calling software used was Sequencing Analysis v5.2. They were then molecularly characterized by 16S rDNA partial gene sequencing based on bioinformatics analysis using BLASTN program.

### Assessment of Co-inoculation

#### Plant Material and Pot Volume

The test crop used was common bean; AFR 708 (Chelalang) and GLP 2 (Rosecoco) varieties. Chelalang is a newly released common bean variety by Egerton University with special attributes of being high yielding, and pest and disease resistant (Njoka et al., 2009). Rosecoco is a high yielding variety suitable for medium altitudes (KALRO, 2008). Nitisols from Chuka in Eastern Kenya were collected from the 0–20 cm top layer, air-dried, sieved to pass 2 mm and thoroughly homogenized. Pots with inner diameter of 15 cm, length of 30 cm, and a volume of 5.3 L were used and contained 4 kg of soil from Chuka. PVC tubes were closed at the bottom using a nylon mesh, and placed on plastic plates.

#### Soil Analysis

The soil from the study-site was characterized (0–20 cm top layer). Soil samples were air-dried, prepared, and analyzed using standard procedures as described by Okalebo et al. (2002). In brief, soil pH was determined using a glass electrode pH meter at 1:2.5 soil/water ratio. Available P was extracted using the Mehlich-3 and determined using the ammonium vanadate method and amount determined using a spectrophotometer (Mehlich, 1984). Organic carbon was determined by Walkley and Black sulfuric acid–dichromate digestion followed by back titration with ferrous ammonium sulfate (Walkley, 1935), whereas nitrogen was determined using the Kjeldahl method (Bremner and Mulvaney, 1982).

#### Treatment Structure for Co-inoculation

The PGPR strains, i.e., *Paenibacillus polymyxa* and *Bacillus megaterium*, isolated and identified in this study, were selected for co-inoculation study based on reports of these strains’ ability to solubilize P in the soil. IITA-PAU 987 and IITA-PAU 983 were rhizobia strains nodulating common bean, recently isolated from Ethiopian soils. The reference strain CIAT 899 (*Rhizobium tropici*) was obtained from the University of Nairobi soil microbiology laboratory. The rhizobia strains were used alone or in co-inoculation with each of the PGPR strains separately (**Table 1**). The experiment was laid out in a CRD with three replicates in the greenhouse and the pots were rotated regularly on the benches to reduce the effect of sunlight intensity at the different times of the day. The positive control contained only N as the focus of the study was on improving the effectiveness of rhizobia strains.

**Table 1:** Treatment structure of the co-inoculation trial.

Factor	Levels	Description of levels
Variety	2	Chelalang, Rosecoco
Soil type	1	Nitisol
Treatments	11	IITA-PAU 983, IITA-PAU 987, CIAT 899, IITA-PAU 983 + *Paenibacillus polymyxa*, IITA-PAU 983 + *Bacillus megaterium*, IITA-PAU 987 + *P. polymyxa*, IITA-PAU 987 + *B. megaterium*, CIAT 899 + *P. polymyxa*, CIAT 899 + *B. megaterium*, positive control (no inoculation + N-fertilizer), negative control (no inoculation, no N-fertilizer)

#### Fertilization Program

Nutrients were added to the experimental pots using the rate described above under Trapping PGPR from the soil, from the standard nutrition solution. For N-control, 100 kg N ha^-1^was applied as 384 kg ha^-1^ Calcium Ammonium Nitrate (CAN = 0.64 g pot^-1^). A quarter (25%) of N (0.16 g) was applied at planting and the remaining 75% (0.48 g) after 3 weeks (Woomer et al., 2011).

#### Inoculum Preparation

For the inoculation study Rhizobia strains were grown in yeast mannitol broth (YMB), in flasks and shaken at 28°C at 200 rpm in a rotary shaker for 5–7 days until (turbid) when logarithmic phase is attained ∼1 × 10^9^cells ml^-1^ (Woomer et al., 2011). Bacillus and *Paenibacillus* strains were cultured in nutrient broth medium in 250 ml flasks and shaken at 200 rpm at 37°C for 24 h. The 5 day old culture of Rhizobia strains and 24 h old cultures of the PGPR strains were used for inoculation (Atieno et al., 2012) as described in **Table 1**.

#### Planting and Inoculation

Seeds were surface-sterilized by soaking in 3.5% NaOCl solution for 5 min and then thoroughly washed with distilled water. Two healthy seeds of uniform size were then planted per pot, and thinned to one plant per pot of comparable height and vigour at 7 days after planting. One milliliter of log phase bacterial culture was inoculated in the treatment pot 7 days after planting. For the co-inoculation treatments, a cocktail consisting of the two strains was prepared in the ratio of 1:1 and 1 ml of the mixture inoculated to the crop 7 days after planting (Wasike et al., 2009). The pots were watered regularly to maintain the soil at field capacity.

#### Data collection

The data collected included nodulation and shoot and root dry weights of the crops. At mid podding after planting, plants were carefully uprooted from the pots and placed on sieves to avoid loss of nodules during cleaning. The soil was then gently washed off the roots under a stream of running tap water. The nodules were then carefully removed from the roots, counted, and weighed. The above ground biomass and root dry weight was taken after drying to a constant weight at 65°C.

The amount of N per plant was estimated using the correlation derived by Mweetwa et al. (2016) relating the number of nodules and the amount of nitrogen accumulated within the common bean plant in an acid soil:

$$\text{Y}=1.9368\text{x}-106.41({\text{R}}^{2}=0.7139);$$

where Y = the number of nodules per plant and X = the amount of tissue nitrogen per plant.

The N-content of shoots (N) was used to calculate amount of nitrogen fixed (Nf) defined as:

Nf=(Ni−Nc)

where i is the inoculated and c is the uninoculated control (Yadegari et al., 2010).

The percent N derived from atmosphere (%Ndfa) was computed according to the following formula:

$$\%\text{Ndfa}=100({\text{TPN}}_{\text{inc}}-{\text{TPN}}_{\text{cont}})/{\text{TPN}}_{\text{inc}}$$

where TPN_inc_ is the total N-content of plants inoculated and TPN_cont_ is the total N-content of uninoculated control (Herridge and Danso, 1995).

#### Data Analyses

Data were collected in replicates of three and analyzed using SAS Statistical Package Version 9.3. To determine the effects due to inoculation, Analysis of Variance at 95% confidence limit was done and means were separated using Duncan Multiple Range Test (DMRT) at α = 0.05. Orthogonal contrasts were also done to test for differences between single and co-inoculation, between CIAT 899 and the other rhizobia strains and between inoculation and the controls.

## Results

### Morphological and Biochemical Characteristics of the Isolated Bacterial Strains

Out of the 19 morphotypes obtained, seven of the colonies showed positive results for gram staining and catalase test. The isolates were found to be first growers (1–2 days). All the isolates produced round shaped and raised colonies having smooth shiny surface with smooth margin. All the isolates were rod shaped (Bacilli) and Gram positive in reaction (**Table 2**).

**Table 2:** Morphological and biochemical properties of the bacteria isolates.

S.No.	Isolate	Colony shape	Color	Elevation	Size	Catalase	Cell shape	Gram stain
1	HK1	Circular	White	Raised	Small	+	Rod	+
2	HK2	Circular	Creamy white	Raised	Medium	+	Rod	+
3	HK3	Circular	White	Raised	Medium	+	Rod	+
4	HK4	Circular	White	Raised	Small	+	Rod	+
5	HK5	Circular	Creamy white	Raised	Small	+	Rod	+
6	HK6	Circular	White	Raised	Small	+	Rod	+
7	HK7	Circular	White	Raised	Medium	+	Rod	+

### Identity of Common Bean Nodule PGPR

Seven PGPR isolates were successfully isolated from the nodules of common bean. They were molecularly characterized by 16S rDNA partial gene sequencing. There was no dominant species within the seven isolates of *Bacillus* sp. Maximum identities for each isolate were between 98 and 99% with *E*-value of 0. The distributions were genetically diverse on several species of *Bacillus* sp., such as *B. megaterium*, *B. subtilis*, *B. aryabhattai*, and *P. polymyxa* (**Table 3**).

**Table 3:** Molecular identification of common bean nodule plant growth promoting rhizobacteria (PGPR) by 16S rDNA sequencing.

Sequence ID	Query length	Blast-related sequence	Accession	*E*-value	Identity
HK1	1447	*Paenibacillus polymyxa*	NC_014483.1	0.0	98%
HK 2	1492	*Bacillus megaterium*	KF658192.1	0.0	98%
HK3	1457	*Bacillus aryabhattai*	KJ009477.1	0.0	98%
HK4	1400	*Bacillus megaterium*	JF496300.1	0.0	99%
HK5	1437	*Bacillus subtilis*	KJ496376.1	0.0	99%
HK6	1441	*Bacillus megaterium*	KC441754.1	0.0	99%
HK7	1427	*Bacillus* sp.	AB508884.1	0.0	98%

### Chemical Properties of the Study Soil

The study soils had a pH of 5.01 (acidic), low level of available P, total nitrogen and organic carbon were moderate (Okalebo et al., 2002; Mungai et al., 2009) (**Table 4**). The soil has been classified as a Rhodic Nitisol (Jaetzold et al., 2005).

**Table 4:** Soil chemical properties of the study soil.

Soil	Depth (cm)	pH (H_2_O)	Available P (mg kg^-1^)	Organic carbon (%)	Total nitrogen (%)
Nitisol	0–20	5.01	14.0	2.46	0.16

### Nodulation of Common Bean

**FIGURE 1:**
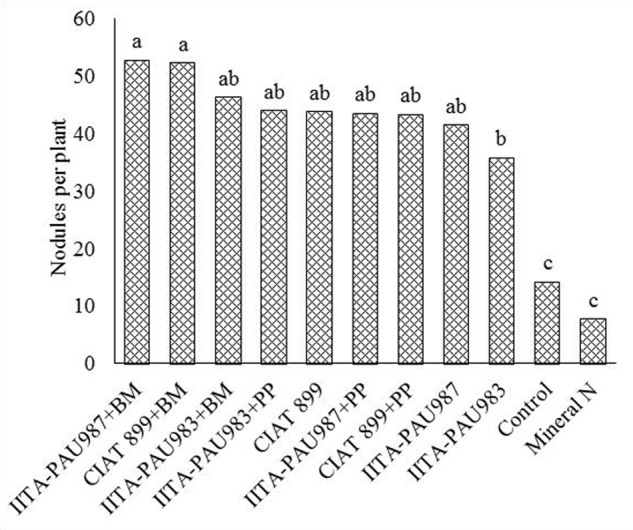
**Effect of co-inoculation on the number of nodules of common bean**. PP, *Paenibacillus polymyxa*; BB, *Bacillus megaterium.* Means followed by the same letter are not significantly different at α = 0.05.

Co-inoculation of the rhizobia strains with the PGPR generally enhanced the nodulation of common bean compared to single rhizobial inoculation. *Bacillus megaterium* + IITA-PAU 987 and *B. megaterium* + CIAT 899 resulted in extra abundant nodulation of the common bean. This was followed by IITA-PAU 983 + *Bacillus megaterium* and IITA-PAU 983 + *P. polymyxa* that recorded abundant nodulation. However, inoculation with CIAT 899 alone recorded a higher nodulation compared to CIAT 899 + *P. polymyxa* and IITA-PAU 987 + *P. polymyxa*. This implies that different PGPR varry in their ability to increase nodulation when co-inoculated with the rhizobia strains with co-inoculation with *B. megaterium* showing higher response than *P. polymyxa*. Inoculation of common beans with rhizobial strains significantly increased the number of nodules of common bean over the control. The rhizobia inoculated treatment had abundant nodulation with the control having moderate nodulation. Application of mineral nitrogen recorded the least (few) number of nodules (**Figure 1**).

### Nodule Fresh Weight, Shoot and Root Dry Weight

Co-inoculation of rhizobia strains with the PGPR increased the nodule fresh weight as compared to single rhizobia inoculation. Co-inoculation of IITA-PAU 987 + *B. megaterium* recorded the highest nodule weight (405.2 mg) compared to IITA-PAU 987 alone (324.8 mg), followed by CIAT 899 + *B. megaterium* (401.2 mg) compared to CIAT 899 alone (337.2 mg). However, CIAT 899 alone recorded a higher nodule weight as compared to CIAT 899 + *P. polymyxa*. Inoculation with rhizobia in combination with the PGPR recorded significantly higher nodule fresh weight compared to the mineral fertilizer application and control. Rhizobia inoculation resulted in a higher nodule fresh weight compared to the control and the mineral nitrogen application (**Table 5**).

**Table 5:** Effect of co-inoculation on the nodule fresh weight, shoot and root dry weight, BNF and %Ndfa of common bean.

Treatment	NFW (mgplant^-1^)	SDW (gplant^-1^)	RDW (gplant^-1^)	BNF	%Ndfa (%)
CIAT 899	337.2ab	5.80cde	0.89ab	15.0ab	18.3ab
CIAT 899 + *B. megaterium*	401.2a	7.23b	1.02ab	15.2ab	19.4ab
CIAT 899 + *P. polymyxa*	331.3ab	5.83cde	0.97abc	19.6a	23.2ab
IITA-PAU 987	324.8ab	5.32e	0.77abc	14.0ab	18.3ab
IITA-PAU 987 + *B. megaterium*	405.2a	6.84bcd	1.01ab	19.9a	24.0a
IITA-PAU 987 + *P. polymyxa*	340.0ab	5.97cde	0.65bc	15.1ab	19.0ab
IITA-PAU 983	268.6b	5.14e	0.65bc	11.1b	14.9b
IITA-PAU 983 + *B. megaterium*	354.8ab	5.70de	0.99ab	15.3ab	19.5ab
IITA-PAU 983 + *P. polymyxa*	336.7ab	7.15bc	0.89ab	16.5ab	20.0ab
Control	137.3c	3.22f	0.45c	–	–
Mineral N	47.2c	9.29a	1.21a	–	–

*Means followed by the same letter within a column are not significantly different at α = 0.05*.

*NFW, nodule fresh weight; SDW, Shoot dry weight; RDW, root dry weight*.

Co-inoculation of rhizobia strains and PGPR recorded a higher shoot dry weight as compared to single rhizobia inoculation. However, this was dependent on the specific rhizobium-PGPR combination. For instance CIAT 899 + *B. megaterium* recorded a significantly (α = 0.05) higher shoot dry weight (7.23 g) compared to CIAT 899 alone (5.80 g). However, there was no significant difference between CIAT 899 + *P. polymyxa* and CIAT 899 alone. Similarly, combination of IITA-PAU 987 and *B. megaterium* led to significantly higher shoot dry weight compared to IITA-PAU 987 alone but no significant difference was observed when co-inoculated with *P. polymyxa*. However, IITA-PAU 983 in combination with *P. polymyxa* led to significantly higher shoot dry weight compared to IITA-PAU 983 alone and IITA-PAU 983 + *B. megaterium*. There was no significant difference among the three rhizobia strains under study on the shoot dry weight of the common bean. However, the three rhizobia strains led to a significant increase in shoot dry weight over the negative control. Mineral nitrogen application recorded the highest shoot dry weight while the control recorded the least (**Table 5**).

In terms of root dry weight, co-inoculation of rhizobia strains with the PGPR did not significantly increase the root dry weight compared to single rhizobial inoculation. Apart from IITA-PAU 983 and IITA-PAU 987 + *P. polymyxa*, all the other inoculation treatments performed at par with the mineral nitrogen application (**Table 4**). Co-inoculation of the rhizobia strains and PGPR significantly increased the root dry weight compared to the control apart from CIAT 899 + *P. polymyxa* and IITA-PAU 987 + *P. polymyxa.* The amount of nitrogen fixed was affected by co-inoculation of Rhizobium with PGPR strains in varieties used in this study (**Table 5**).

In terms of N fixation, there was no significant difference between the rhizobial strains and the reference commercial strain (CIAT 899). Inoculation of common bean with IITA-PAU 983 recorded significantly less nitrogen (11.1) compared to the co-inoculation of IITA-PAU 987 with *B. megaterium* (19.9) and co-inoculation of CIAT 899 with *P. polymyxa* (19.9) indicating that these PGPR has promising effect on enhancement of symbiotic performance of rhizobial strains. Percentage of nitrogen derived from atmosphere (%Ndfa) also followed a similar trend as of amount of nitrogen fixed (**Table 4**). For example, plants inoculated with IITA-PAU 987 and *B. megaterium* received 24.0 % of their nitrogen demand from atmosphere, which showed a 31.1% increase compared to rhizobium alone (**Table 5**).

### Selected Orthogonal Contrasts

Based on contrast analysis, rhizobia inoculation resulted in higher shoot and root dry weights compared to control treatments (**Table 5**). Similarly, co-inoculated plants resulted in higher shoot and root dry weights, BNF and %NDFA than single rhizobial inoculation. This implies that there is an advantage of co-inoculation over single rhizobia inoculation on the growth parameters of common bean. However, contrast analysis did not show any differences between inoculation of common bean with either rhizobia alone and in combination with PGPR, and the application of mineral nitrogen at the rate of 100 kg N ha^-1^. Similarly, no differences were observed between the reference strain (CIAT 899) and the two rhizobia strains (IITA-PAU 983 and IITA-PAU 987) in all the parameters measured showing that the two strains had comparable effect on the shoot and root dry weights of common bean (**Table 6**).

**Table 6:** Mean square table of orthogonal contrasts of various treatment combinations on shoot and root dry weights and biological nitrogen fixation of common bean.

Contrast	Shoot dry weight	Root dry weight	BNF	%NDFA
Rhizobia inoculation vs. Co-inoculation	110.5^∗∗∗^	1.7^∗∗∗^	4.3^∗∗^	4.1^∗∗^
CIAT 899 vs. IITA-PAU 983 and IITA-PAU 987	0.7^ns^	0.2^ns^	0.6^ns^	0.7^ns^
Inoculation vs. +Nitrogen	5.0^ns^	0.01^ns^	–	–
Inoculation vs. control	0.3^∗^	0.1^∗^	–	–
Control vs. others	75.6^∗∗∗^	1.5^∗∗∗^	–	–

*%NDFA, percent of nitrogen derived from the atmosphere; BNF, Biological nitrogen fixation*.

*^∗^, significant at 0.05 level; ^∗∗^, significant at 0.01 level; ^∗∗∗^, significant at 0.001 level; ns, not significant*.

## Discussions

Non-rhizobial root endophytes were isolated from root nodules of common bean. An increasing number of α, β, and γ_*Proteobacteria* have been isolated from root nodules of a wide range of legumes and are reported as nodule associated bacteria or nodule endophytes (Bai et al., 2002; Zakhia et al., 2006; Kan et al., 2007). Such nodule associated bacteria may be endophytic or free living rhizobacteria and may establish neutral or beneficial interactions with plants (Li et al., 2008; Muresu et al., 2008; Stajkovic, 2009; Pandya et al., 2015). Results from this study are in line with the studies by Rajendran et al. (2008) who reported the presence of high proportion of gram positive endophytes within the root nodules of pigeon pea.

Root nodules accommodate various non-nodulating bacteria having definite influence on the survival, nodulation, and grain yield of crop (Mishra et al., 2009; Tariq et al., 2014). Hung et al. (2007) reported the isolation of *Paenibacillus polymyxa* HKA-15, a Gram-positive bacterium from root nodules of soybean. A total of 75 endophytic bacteria isolated from nodules of field pea and 88 from nodules of chickpea showed that 93.4% in were Gram positive (Narula et al., 2013). Diep et al. (2016) also isolated endophytic bacteria in soybean root nodules.

Considering that some PGPR possess ability of phosphate solubilization, they could be useful in bean production improvement by increasing P content in the soil and enhancing nodulation and N fixation. The inoculation of plants with PGPR can increase native population through various mechanisms that convert insoluble inorganic and organic soil P into plant available forms and therefore improve plant nutrition (Guiñazu et al., 2010; Qureshi et al., 2012; Sharma et al., 2013; Singh, 2013). So, PGPR has enormous potential in biofertilizer formulations to be exploited in increasing crop yields by solubilizing fixed P in the soil (Hayat et al., 2012).

Co-inoculation of *Rhizobium* with *B. megaterium* and *P. polymyxa* enhanced the shoot and root dry weights as compared to *Rhizobium* inoculation alone. This may be due to direct and indirect enhancement of plant growth by a variety of mechanisms such as production of growth promoting substance and solubilization of minerals such as P (Verma et al., 2012). Co-inoculation, frequently, increase growth and yield, compared to single inoculation, provided the plants received balanced nutrition, and improved absorption of nitrogen, phosphorus, and mineral nutrients (Araújo et al., 2009). Phosphorous availability increases the number and size of nodules and the amount of nitrogen assimilated per unit weight of nodules, increasing the percent and total amount of nitrogen in the harvested portion of the host legume and improving the density of Rhizobia bacteria in the soil surrounding the root (Bashir et al., 2011). Phosphorous deficiency has been shown to affect symbiosis by decreasing the supply of photosynthates to the nodule, which reduces the rate of bacterial growth and the total population of legume-nodulating microorganisms (Moreira et al., 2010). The positive effect of combined inoculation of endophytic bacteria with *Rhizobium* spp. can be attributed to an early nodulation, an increase in the number of nodules, or a general improvement in root development (Sa et al., 2012).

The results from this study showed increased number of nodules and nodule weight due to co-inoculated with *P. polymyxa* and *B. megaterium* in respect to the inoculation with *Rhizobium* alone in common bean. Similarly, Stajkovic et al. (2011) reported that co-inoculation of bean with *Rhizobium* + *Bacillus* strains SNji and *Rhizobium* + *Bacillus* strains Bx positively influenced nodule number (106.67 and 76.67 nodule number plant^-1^, respectively) compared to inoculation with *Rhizobium* alone (54 nodule number plant^-1^). Enhancement in nodule number, nodular mass due to combined inoculation might be the expansion in root length and mass, thus more number of active sites for nodulation by the rhizobial strains.

Co-inoculation of common bean with *Rhizobium* and the PGPR, significantly increased shoot and root dry weights of plants in respect to *Rhizobium* inoculation alone and the uninoculated control showing the potential of these strains to improve dry matter accumulation over *Rhizobium* alone. Similarly, Figueiredo et al. (2008) recorded a higher shoot and root dry weight when CIAT 899 rhizobia strains were co-inoculated with *Paenibacillus polymyxa* strain DSM 36 than single inoculation with CIAT 899 strain in common bean. Additionally, Elkoca et al. (2010) reported an increased shoot dry weight as a result of co-inoculation of common bean with *Bacillus megaterium* (M-3) strain and *Rhizobium* strain. In studies with other legumes, synergism between Bacillus and *Bradyrhizobium* in the rhizosphere has been shown to increase nodulation and plant biomass (Tilak et al., 2006; Wani et al., 2007; Medeot et al., 2010; Tsigie et al., 2012). In soybean, pot experiments were conducted by Fatima et al. (2006) to evaluate the effects of *Rhizobium leguminosarum* strain alone and in combination with PGPR. The plants grown with combination showed increased root and shoot weight suggesting a promising way for enhancing the growth of legume crops. The results from field and greenhouse chickpea experiments by Verma et al. (2012) showed significant results with the combination of *Mesorhizobium* sp. BHURC02 with *B. megaterium*, to be superior over uninoculated control. Results from the contrast analysis showed that co-inoculation of common bean with rhizobia strain and the two PGPR strains were comparable to mineral nitrogen addition. Therefore these strains can be used in the formulation of agricultural product and provide cheaper alternative to farmers in production of common bean in such soils.

Positive effects of inoculation of common bean with appropriate rhizobia strains have been widely reported. Gicharu et al. (2013) reported a significant increase in nodulation when three climbing bean cultivars were inoculated with CIAT 899 compared to the control treatments. Similar results on the effect of inoculation compared to control on nodulation, shoot and root dry weights have been reported (Javaid and Mahmood, 2010; Mehrpouyan, 2011; Trabelsi et al., 2011). The comparable effect of CIAT 899 as a reference strain with the other two strains in this study shows a possibility of additional options in exploiting elite local strains adapted to local conditions. The strains can be used in product formulation to give farmers an alternative to the expensive inorganic fertilizers and help mitigate environmental issues raised by the excessive use of inorganic fertilizers.

## Conclusion

The present study showed the presence of endophytic non-rhizobial bacteria in common bean nodules. They belonged to the most common genera connected with PGPR, i.e., Bacillus indicating a degree of coexistence of the PGPR and rhizobia within the nodules. Co-inoculation of rhizobia strains with the tested PGPR enhances the growth of common bean in a phosphorous deficient soil. Therefore, *P. polymyxa* and *B. megaterium* strains can be used together with the tested rhizobia strains to improve growth of common bean in soils that are low in phosphorous. *Bacillus megaterium and P. polymyxa* strains can be used in further investigations as potential agents of new biofertilizer formulations for improved common bean production. Inoculation of common bean with the two rhizobia strains, i.e., IITA-PAU 983 and IITA-PAU 987 strains performed at par with inoculation with the reference strain, CIAT 899, in enhanced nodulation, shoot and root dry weight over the control treatment and, therefore, offer alternative options for rhizobia inoculants for common beans. Further studies under field conditions and at multiple locations will be needed to corroborate the findings of this study.

## Author Contributions

HK is a student taking Masters in Soil Science at Egerton University, Kenya. He did the lab and greenhouse trials, data analysis and the first draft of the manuscript. Part of the lab work was done at Egerton University and the other at IITA lab in Nairobi. NM is a supervisor based at Egerton University, she coordinated and supervised the work, assisted in data analysis and interpretation, and editing of the manuscript. MT is a supervisor based at IITA, Nairobi. He supervised the molecular analysis of section of the work, assisted in data analysis and interpretation and editing of the manuscript. YH isolated and characterized the native strains IITA-PAU 987 and IITA-PAU 983. CM is a supervisor based at IITA and the COMPRO II project leader. He co-supervised the molecular work at IITA and edited the manuscript.

## Conflict of Interest Statement

The authors declare that the research was conducted in the absence of any commercial or financial relationships that could be construed as a potential conflict of interest.
